# AI-Enabled Telerehabilitation for Chronic Nonspecific Neck Pain: Multicenter Randomized Controlled Trial

**DOI:** 10.2196/87487

**Published:** 2026-10-15

**Authors:** Yiwei Zhao, Yanbin Zhang, Ye Lin, Di Liu, Jiamin Zheng, Siyi Cai

**Affiliations:** 1 Department of Orthopedics, Chinese Academy of Medical Sciences & Peking Union Medical College, No.1 Shuaifuyuan, Dongcheng District, Beijing, China, 86 010-69151188; 2 Department of Spine Surgery, Beijing Jishuitan Hospital, Capital Medical University, Beijing, China; 3 Pritzker School of Medicine, University of Chicago, Chicago, IL, United States; 4 Jiakang Zhongzhi Technology Co, Beijing, Beijing, China

**Keywords:** chronic nonspecific neck pain, telerehabilitation, physical therapy, smartphone sensors, randomized controlled trial

## Abstract

**Background:**

Exercise therapy is effective in managing chronic nonspecific neck pain (CNP). Nevertheless, the high burden of CNP continues to place pressure on existing rehabilitation services, especially in remote or resource-constrained settings where continuous and standardized physical therapy may be difficult to access. By combining home-based exercise with remote supervision and movement feedback, AI-enabled telerehabilitation may broaden access to rehabilitation and support the continued management of CNP.

**Objective:**

This study examined whether an AI-enabled telerehabilitation program was noninferior to standard outpatient rehabilitation for improving neck-related disability in individuals with CNP and compared the effects of the 2 interventions on pain, health-related quality of life, and cervical range of motion.

**Methods:**

This multicenter, prospective, parallel-group randomized noninferiority trial was conducted at Peking Union Medical College Hospital and Beijing Jishuitan Hospital. Patients with CNP of at least 3 months’ duration were randomized 1:1 to a telerehabilitation group (TG) or outpatient rehabilitation group (OG) using concealed, web-based, variable blocks stratified by center and baseline Neck Disability Index (NDI) severity. Both groups completed identical 24-week strengthening and stretching programs (3 sessions per wk; ≈30 min per session) and were assessed at weeks 0, 4, 8, 12, and 24. TG trained at home using sensor-based movement recognition and threshold rules; OG received face-to-face outpatient rehabilitation. Week-24 NDI was the primary outcome; Numeric Pain Rating Scale (NPRS), EQ-5D, and cervical range of motion were secondary. Noninferiority required the upper 95% CI bound of the TG-OG NDI difference to be <10 percentage points (5/50). Analyses were intention-to-treat; assessors and statisticians were blinded.

**Results:**

Of 312 individuals screened, 116 were randomized (58 per group). At week 24, 55 TG and 53 OG participants completed assessments; all 116 participants were included in the primary intention-to-treat analysis using observed or imputed data. The between-group differences in NDI (TG − OG) were −2.8 (95% CI −6.3 to 0.7; *P*=.11) at week 12 and −2.5 (95% CI −6.2 to 1.2; *P*=.15) at week 24. At week 24, the upper 95% CI bound was below the prespecified noninferiority margin of 10 percentage points on the 0 to 100 NDI scale, equivalent to 5 points on the original 0 to 50 scale, demonstrating noninferiority of TG. Both groups showed clinically meaningful reductions in NDI, with comparable improvements in NPRS, EQ-5D, and cervical range of motion. No intervention-related harms occurred.

**Conclusions:**

Over 24 weeks, AI-enabled telerehabilitation was noninferior to exercise-matched outpatient rehabilitation for neck-related disability, with broadly comparable improvements in pain, health-related quality of life, and cervical range of motion. AI-enabled telerehabilitation may be a practical alternative for patients with CNP who are unable to attend outpatient rehabilitation.

## Introduction

### Background and Rationale

Chronic nonspecific neck pain (CNP) refers to pain lasting more than 12 weeks that is localized to the lateral and posterior neck without evidence of a specific underlying pathology [1,2]. CNP is among the most common musculoskeletal disorders worldwide and is associated with substantial disability, reduced quality of life, impaired work productivity, and considerable socioeconomic burden [3,4]. According to estimates from the Global Burden of Disease 2021 study, the age-standardized prevalence of neck pain is approximately 2450 per 100,000 population, and the age-standardized rate of years lived with disability is approximately 244 per 100,000, corresponding to approximately 203 million prevalent cases worldwide [4-7].

In China, the burden of neck pain has continued to rise since 1990, reaching approximately 68 million prevalent cases in 2019 [8-11]. The size of this patient population, together with the persistent disability caused by neck pain, has increased the need for physical therapy and ongoing rehabilitation services, placing further strain on existing rehabilitation resources. Effective, sustainable, and scalable approaches are therefore required for the long-term management of CNP.

Exercise therapy is among the best-supported interventions for CNP. Structured exercise programs can relieve pain, improve physical function, and lead to favorable clinical outcomes [12]. These programs commonly include strengthening, stretching, motor control, and postural exercises tailored to the patient’s symptoms and functional capacity. Maintaining exercise quality over time requires appropriate instruction, monitoring, and adjustment as symptoms and functional status change. Continued participation in standardized strengthening and stretching exercises is therefore an important part of long-term CNP management.

In the traditional outpatient rehabilitation model, patients attend structured exercise programs regularly under the ongoing supervision of physical therapists. However, access to physical therapy and rehabilitation services is often limited or interrupted for those living in remote or resource-constrained areas. In China, population aging and the growing burden of chronic disease have further increased the demand for rehabilitation, while shortages of rehabilitation professionals and regional imbalances in workforce distribution persist [13-17]. Travel difficulties, work-related commitments, and repeated hospital visits may also contribute to delayed care, missed appointments, incomplete treatment, and poorer adherence [18-21].

Consequently, the traditional outpatient model may not fully meet the needs for continuous rehabilitation in patients with CNP. Face-to-face care usually allows only intermittent observation, which limits the continuous monitoring of home exercises, real-time correction of compensatory movements, and timely adjustment of exercise prescriptions when appointments are missed. Telerehabilitation may help address these limitations by integrating home-based exercise into a remotely supervised rehabilitation program. This approach can improve access, lessen the burden of travel, and maintain continuity of care [21-23]. In an AI-enabled model, real-time feedback can support correct exercise performance, sensor-based monitoring can detect movement deviations and deliver corrective feedback, and recorded training data can guide timely therapist-led adjustments to the exercise program.

In recent years, various telerehabilitation strategies have been examined for chronic musculoskeletal pain, including smartphone-delivered exercise guidance, sensor-assisted movement monitoring, digital training logs, real-time feedback, and remote supervision by therapists. Evidence from systematic reviews, scoping reviews, meta-analyses, and randomized controlled trials generally indicates that telerehabilitation can reduce chronic musculoskeletal pain and improve physical function [21-24]. Collectively, these findings suggest that digital technologies can bring standardized exercise programs and professional supervision into patients’ homes.

However, few clinical trials have directly compared telerehabilitation with standard outpatient rehabilitation in patients with CNP, particularly over intervention periods of 12 weeks or longer. Existing studies also vary substantially in their comparator interventions, treatment durations, digital functions, and the degree of therapist supervision [21-24]. This heterogeneity makes it difficult to determine whether remotely delivered structured exercise can preserve the effects of exercise-matched face-to-face care over a longer period. Systematic evaluations of sensor-supported or AI-enabled home-based programs are also scarce, and evidence remains limited in settings such as China, where rehabilitation demand substantially exceeds the available workforce capacity [21-24].

Beyond neck-related disability, the effectiveness of treatment for CNP should also be evaluated in terms of pain intensity, health-related quality of life, and cervical range of motion (ROM). These measures respectively capture symptom severity, general health status, and functional mobility of the cervical spine, allowing a broader assessment of rehabilitation outcomes [2,25-29]. Rigorous clinical evaluation is therefore required to establish whether AI-enabled telerehabilitation produces outcomes comparable to standard outpatient rehabilitation and whether it offers a practical, scalable option for the long-term management of CNP in resource-constrained settings.

### Objectives

This study evaluated the Healbone Rehabilitation System (HRS; Jiakang Zhongzhi Technology Co), which combines a smartphone-based platform, integrated sensors, AI-assisted movement feedback, rule-based exercise support, and remote therapist supervision. The primary objective was to assess whether the intervention was noninferior to standard outpatient rehabilitation in reducing neck-related disability among patients with CNP, using the Neck Disability Index (NDI) at week 24 as the primary outcome. During the structured home-based exercise program, the system provided guidance and monitored patient performance to support adherence and continuity of care. The effects of the 2 interventions on pain, health-related quality of life, and cervical ROM were also compared using the Numeric Pain Rating Scale (NPRS), EQ-5D, and cervical ROM assessments, respectively.

## Methods

### Ethical Considerations

Ethical approval was granted by the Ethics Committee of Peking Union Medical College Hospital (approval number: I-25PJ0723; March 24, 2025) and the Ethics Committee of Beijing Jishuitan Hospital (approval number: [2025]337‐00).

The protocol was completed before recruitment started on March 25, 2025. Its trial design, eligibility criteria, prespecified primary and secondary outcomes, and planned primary statistical analyses were consistent with the information retrospectively entered in the Chinese Clinical Trial Registry. None of these elements were changed after the trial began.

The trial was registered retrospectively in the Chinese Clinical Trial Registry (ChiCTR2500112693) on November 18, 2025, after participant enrollment had started. Prospective registration had been unintentionally omitted during trial preparation because of an administrative oversight, rather than an attempt to circumvent trial registration.

To address the possibility of selective reporting, the ethics-approved protocol was established before the first participant was recruited. The study design, eligibility requirements, prespecified primary and secondary outcomes, and planned primary analyses remained unchanged during the trial.

All procedures followed the principles of the Declaration of Helsinki and the International Council for Harmonisation Good Clinical Practice guidelines. Before randomization, participants were informed of the study purpose, procedures, possible risks, and expected benefits and then provided written informed consent. They were also told that participation was voluntary and that they could leave the study at any time without giving a reason or facing any penalty. Study data were deidentified, stored securely, and made accessible only to authorized research staff to safeguard privacy and confidentiality. All images in the paper and Multimedia Appendix 1 were deidentified to ensure that no individual participant or user could be identified. No financial compensation was provided, although the intervention and all study-related procedures were offered free of charge. The trial results have not been publicly posted or published elsewhere.

### Patient and Public Involvement

Patients and members of the public were not involved in the design, conduct, or reporting of this trial.

### Trial Design

This study was a multicenter, prospective, 2-arm, parallel-group randomized noninferiority trial with blinded outcome assessment and statistical analysis, conducted at Peking Union Medical College Hospital and Beijing Jishuitan Hospital. Participants were randomized in a 1:1 ratio to the telerehabilitation group (TG) or the outpatient rehabilitation group (OG). All participants were assessed for neck-related disability, pain, health-related quality of life, and cervical ROM at baseline (week 0) and weeks 4, 8, 12, and 24. This study was prepared in accordance with the CONSORT (Consolidated Standards of Reporting Trials) 2025 statement (Checklist 1) [30] and the CONSORT-EHEALTH (Consolidated Standards of Reporting Trials of Electronic and Mobile Health Applications and Online Telehealth Interventions) guidelines (Checklist 2) [31].

### Changes to Trial Protocol

The ethics-approved trial protocol remained unchanged after participant recruitment began. The responder-based analyses were exploratory, undertaken post hoc, and not included in the original analysis plan. Participants became aware of their group allocation after randomization because they needed to follow the assigned rehabilitation procedures. Treating physical therapists were also aware of the allocation, whereas outcome assessors and statistical analysts remained blinded. Therefore, the registry statement resulted from an error during retrospective registration rather than a change in trial procedures.

### Trial Setting

Participants were recruited from the orthopedic and rehabilitation medicine outpatient clinics at 2 hospital sites in Beijing, China: Peking Union Medical College Hospital and Beijing Jishuitan Hospital.

### Eligibility Criteria

At both centers, physicians screened patients with suspected CNP, excluded red-flag conditions, and referred potentially eligible patients to the study coordinator for eligibility confirmation. Eligible patients were enrolled after providing written informed consent. During follow-up, participants were asked not to initiate new neck pain–related treatments outside the study protocol unless clinically necessary, and any treatment changes were documented. The full inclusion and exclusion criteria are presented in Textbox 1.

Textbox 1. **Inclusion and exclusion criteria.**

**Inclusion criteria**

- Age: 18-60 years
- Condition: chronic nonspecific neck pain for ≥3 months
- Baseline Neck Disability Index ≥15/50 and Numeric Pain Rating Scale (7-day average) ≥3/10
- Able to participate in exercise-based rehabilitation
- Able to use a smartphone and willing to use the digital system

**Exclusion criteria**

- Prior cervical spine surgery and major cervical trauma or radiologic instability within the past 12 months
- Progressive neurologic deficit or myelopathy; severe canal stenosis or lesions requiring surgery; active infection, tumor, or acute instability
- Inflammatory spondyloarthropathy; moderate-to-severe congenital or acquired deformity; symptomatic neural compression
- Uncontrolled major organ disease; severe psychiatric illness or cognitive impairment compromising adherence
- Pregnancy or lactation
- Cervical-related rehabilitation or physical therapy or interventional analgesia within 3 months before enrollment

### Intervention and Comparator

#### Overview

Both the TG and OG followed the same strengthening and stretching program to improve neck-shoulder stability, coordination, and postural control. Training was performed 3 times weekly for approximately 30 minutes over 24 weeks, with standardized progression during weeks 0‐4, 4‐12, and 12‐24. The detailed program is provided in Multimedia Appendix 1. Before the trial, all physical therapists received standardized training on progression, regression, and documentation.

#### TG

Participants assigned to the TG completed home-based rehabilitation using the HRS. This smartphone-based telerehabilitation platform combines sensor-assisted movement monitoring with standardized exercise guidance, real-time feedback, and remote supervision by therapists. Both the system and the accompanying intervention were available at no cost. Figure 1 illustrates participants wearing the sensor while receiving exercise instructions and feedback through a smartphone.

**Figure 1.**
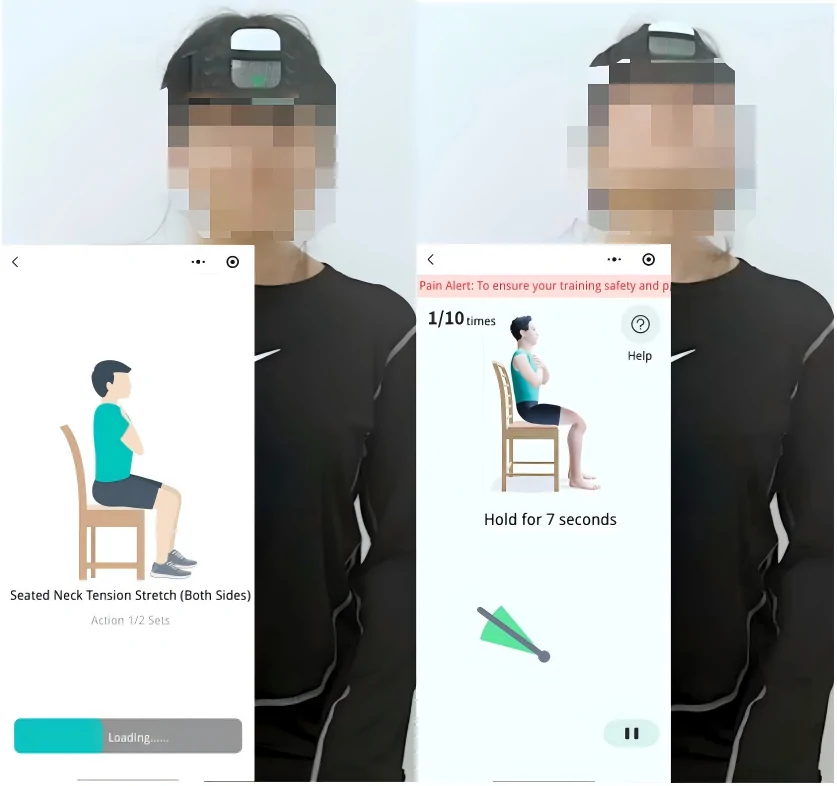
Schematic diagram of the telerehabilitation platform. Participants performed home-based rehabilitation using smartphone-based guidance, sensor-based monitoring, and remote therapist supervision.

The exercise content, prescribed movement patterns, and criteria for progression and regression were established before the intervention began. At enrollment, participants received account-registration support and standardized instructions on platform use and exercise safety. Prescribed exercises were delivered through the platform, and participants calibrated the sensor before each session. If calibration failed, recalibration was required before training. Only sensor data collected after successful calibration during prescribed exercise sessions were processed by the system. If a scheduled session was missed, the platform issued a reminder and notified the responsible physical therapist for follow-up when needed. Participants could also contact the physical therapist through the platform with questions about neck pain symptoms or exercise performance.

During training, participants followed the movement demonstrations and standardized instructions provided through the platform. HRS version 1.0 was used throughout the trial. The system used movement-recognition and threshold-based decision logic to provide real-time feedback and identify incomplete movements or substantial deviations from the predefined patterns. Based on recorded performance, the platform also generated rule-based recommendations for progression or adjustment. Physical therapists reviewed the training data and recommendations, made the final decisions according to prespecified criteria, and delivered the adjusted exercise program. No formal performance-error analysis was planned because the system did not make autonomous decisions.

During the 24-week intervention, the exercise prescription, dosage, and phase-based progression were identical to those in the OG. Participants trained at home 3 times per week for approximately 30 minutes per session, while the platform recorded exercise completion and performance. Scheduled questionnaires were completed through the platform at baseline and at weeks 4, 8, 12, and 24.

#### OG

Participants in the OG completed the same exercise program, dosage, and progression and regression criteria as the TG under face-to-face physical therapist supervision. They attended 3 sessions per week for approximately 30 minutes over 24 weeks and completed outpatient assessments at baseline and at weeks 4, 8, 12, and 24. Both groups followed the same exercise frequency, phase-based progression, and missed-session follow-up procedures; adherence was documented using attendance and prescription-completion records in the OG and platform-generated training logs in the TG.

### Outcomes

The NDI was the primary outcome, with week 24 prespecified as the primary end point and week 12 as an intermediate inferential time point. The NDI is a validated measure of neck-related disability [25]. Scores were converted to a 0 to 100 percentage scale, with higher scores indicating greater disability. Previous studies have established an NDI minimal clinically important difference (MCID) of approximately 5 to 7 points on the original 0 to 50 scale, equivalent to 10 to 14 percentage points on the 0 to 100 scale [26]. Thus, a reduction of at least 5 points on the original scale, equivalent to 10 percentage points on the 0 to 100 percentage scale, was considered a clinically meaningful improvement.

Secondary outcomes included pain intensity, health-related quality of life, and cervical active ROM. Pain was assessed using the NPRS, recorded as the average pain over the previous 24 hours on a 0 to 10 scale. Health-related quality of life was assessed using the EQ-5D-5L, with the Chinese value set used to calculate the utility index. Cervical ROM was measured by trained assessors using a goniometer for flexion-extension, lateral flexion, and rotation, with the mean of 2 trials per movement plane used for analysis. Previous studies suggest MCIDs of approximately 2 points or a 30% reduction from baseline for the NPRS [27], 5° to 10° for cervical ROM, depending on the movement plane and baseline limitation [2], and 0.03 to 0.07 for the EQ-5D utility index [28,29].

All outcomes were assessed at baseline and at weeks 4, 8, 12, and 24. Weeks 12 and 24 were prespecified inferential time points, whereas weeks 4 and 8 were used to describe the trajectory of change during follow-up. Participants in the TG completed questionnaire-based assessments through the HRS platform, whereas those in the OG completed the same questionnaires under outpatient supervision.

### Harms

During the 24-week intervention, participant-reported symptom worsening or other exercise-related adverse events were documented by the treating physical therapists in both groups.

### Sample Size

Sample size estimation was performed using PASS (version 22.0; NCSS Corp) within a 2-sided noninferiority framework, with the week-24 NDI score designated as the primary outcome. The noninferiority margin was set in advance at 10 percentage points on the 0 to 100 NDI scale, corresponding to 5 points on the original 0 to 50 scale. Published studies have reported an NDI MCID of approximately 5 to 7 points on the original scale; accordingly, 5 points, the lower end of this range, was chosen as the largest clinically acceptable difference between groups [25,26]. With an assumed SD of 15 percentage points, 90% power, and a 2-sided α of .05, 48 participants were needed in each group. Allowing for 20% attrition and 1:1 allocation, the target sample was 58 participants per group, or 116 in total. Given the low-risk nature of this rehabilitation trial, no interim analyses were planned; the trial could be stopped for serious safety concerns or at the request of the ethics committees or participating institutions.

### Randomization

All 116 participants were randomly assigned in a 1:1 ratio to the TG (n=58) or the OG (n=58). Independent statisticians prepared a variable block randomization sequence stratified by study center and baseline NDI severity. Group assignments were concealed in a secure web-based system until the baseline assessment had been completed. Personnel responsible for enrollment had no access to the allocation sequence.

### Blinding

Participants and treating therapists knew their assigned group, although participants were not informed of the specific noninferiority hypothesis or whether either intervention was expected to perform better. Outcome assessors and statistical analysts remained blinded throughout the trial.

### Statistical Methods

The outcomes were analyzed according to the intention-to-treat principle, and all randomized participants were analyzed according to their original group assignments. Missing data were assessed using Little’s missing completely at random (MCAR) test and handled using multiple imputation by chained equations.

All analyses were performed using SPSS (version 29.0; IBM Corp). Continuous variables are presented as means (SDs), and categorical variables are presented as frequencies (percentages). Baseline characteristics were compared using 2-sided independent-samples *t* tests or chi-square tests, as appropriate. For the exploratory post hoc responder analyses, between-group responder proportions were compared using 2-sided Fisher exact tests. Continuous outcomes are presented as between-group mean differences with 95% CIs, and the primary NDI outcome was compared with the prespecified noninferiority margin. Statistical analysts remained blinded to group allocation.

## Results

### Participant Flow, Recruitment, and Numbers Analyzed

Between March and October 2025, 312 patients were screened across the 2 centers. Of these, 196 were excluded, including 142 who did not meet the eligibility criteria, 39 who declined participation, and 15 who were excluded because of withdrawal before randomization, incomplete baseline assessment, or other documented reasons. The remaining 116 participants were randomized in a 1:1 ratio to the TG (n=58) or the OG (n=58), as shown in Figure 2.

**Figure 2.**
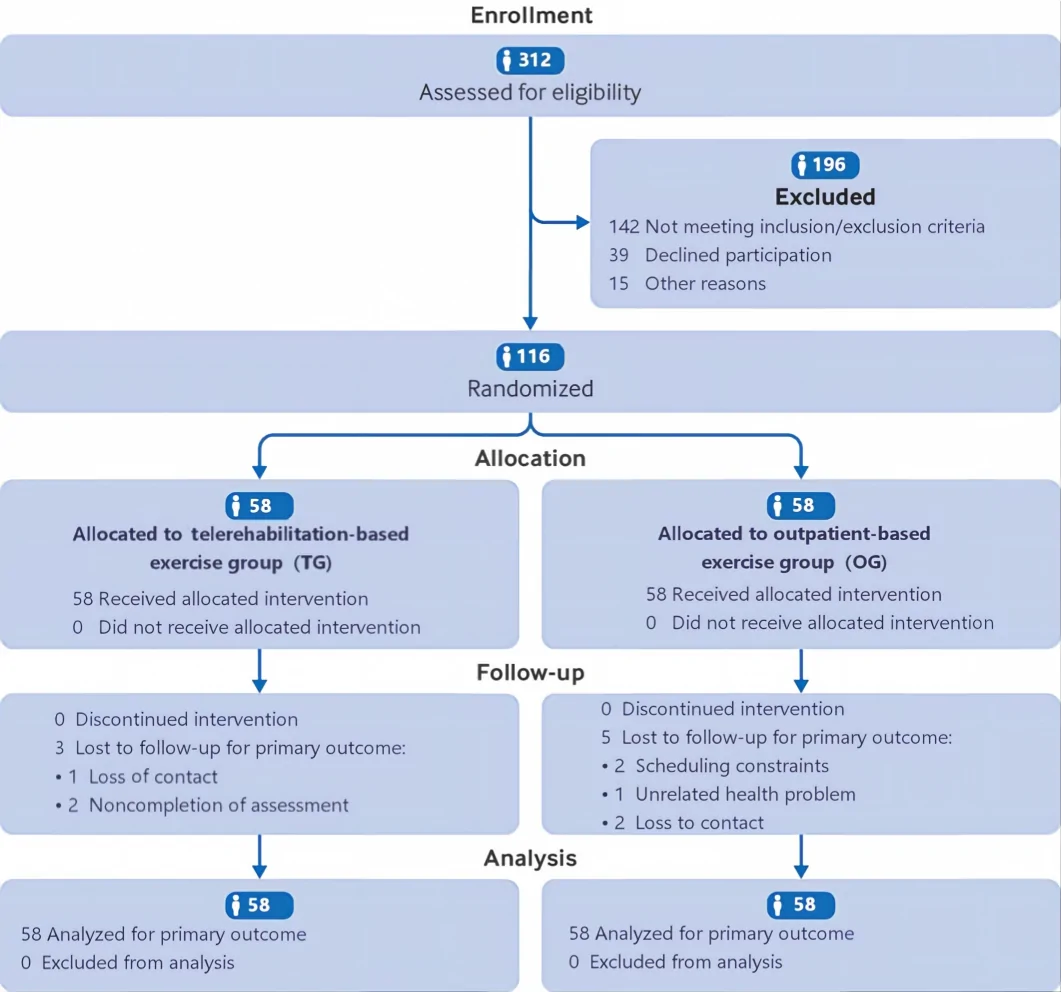
Flowchart of participant enrollment, randomization, allocation, follow‑up, and analysis.

All participants completed the baseline assessment and were scheduled for follow-up at weeks 4, 8, 12, and 24. At week 24, 55 (94.8%) participants in the TG and 53 (91.4%) in the OG completed the assessment. Three participants in the TG did not complete follow-up because of loss of contact or noncompletion of the assessment, whereas 5 in the OG did not complete follow-up because of scheduling constraints, an unrelated health problem, or loss of contact. Thus, participant-level missingness at week 24 was 6.9% (8/116) overall (TG: 5.2%, 3/58 and OG: 8.6%, 5/58). No item-level data were missing from completed questionnaires or assessments (0%). Little’s MCAR test did not reject the MCAR assumption (*χ*²_36_=33.1; *P*=.61).

### Intervention and Comparator Delivery and Baseline Data

No participants in either group received additional neck pain–related treatment during the trial. Baseline demographic and clinical characteristics were similar between the groups (Table 1).

**Table 1.** Participant demographics and clinical characteristics.

Characteristics	OG^a^ (n=58)	TG^b^ (n=58)	*P* value
Age (y), mean (SD)	39.7 (11.5)	40.1 (11.0)	.84
Sex, n (%)			
Male	16 (28)	17 (29)	.84
Female	42 (72)	41 (71)	.86
BMI (kg/m²), mean (SD)	24.8 (5.1)	25.2 (5.3)	.68
Education ≥10 y, n (%)	40 (69)	41 (71)	.84
NDI^c^ (%), mean (SD)	40.9 (11.2)	41.2 (10.9)	.88
NPRS^d^, mean (SD)	5.6 (1.6)	5.9 (1.4)	.28
EQ-5D, mean (SD)	0.69 (0.12)	0.71 (0.14)	.41
ROM^e^ (°), mean (SD)			
Flexion	51.9 (7.9)	52.1 (8.1)	.89
Extension	54.8 (10.1)	55.0 (9.9)	.91
Left lateral flexion	36.4 (8.2)	38.5 (9.3)	.20
Right lateral flexion	37.5 (9.4)	36.6 (7.2)	.56
Left rotation	55.4 (7.5)	53.3 (6.4)	.11
Right rotation	56.0 (7.6)	55.1 (7.4)	.52

a OG: outpatient rehabilitation group.

b TG: telerehabilitation group.

c NDI: Neck Disability Index.

d NPRS: Numeric Pain Rating Scale.

e ROM: range of motion.

### Outcomes and Estimation

#### Primary Outcome

Baseline NDI percentage scores were comparable between the OG and TG, with mean scores of 40.9% (SD 11.2%) and 41.2% (SD 10.9%), respectively (Table 1). In the OG, the mean NDI score was 26.9% (SD 10.1%) at week 4, 22.5% (SD 9.7%) at week 8, 21.9% (SD 9.6%) at week 12, and 20.8% (SD 9.2%) at week 24. In the TG, the mean NDI score was 24.1% (SD 9.8%) at week 4, 20.2% (SD 9.3%) at week 8, 19.1% (SD 9.3%) at week 12, and 18.3% (SD 10.8%) at week 24. At week 24, mean NDI scores in both groups had decreased by more than 10 percentage points from baseline, equivalent to 5 points on the original 0 to 50 scale, indicating clinically meaningful improvement (Table 2).

At week 4, the between-group difference in NDI score (TG − OG) was −2.8 (95% CI −6.5 to 0.9 percentage points; *P*=.13). At week 8, the difference was −2.3 (95% CI −5.8 to 1.2 percentage points; *P*=.20). At week 12, the difference was −2.8 (95% CI −6.3 to 0.7 percentage points; *P*=.11). At week 24, the difference was −2.5 (95% CI −6.2 to 1.2 percentage points; *P*=.15). No statistically significant between-group differences were observed at any follow-up time point. At the primary week-24 end point, the upper bound of the 95% CI was 1.2 percentage points, below the prespecified noninferiority margin of +10 percentage points, demonstrating that the TG was noninferior to the OG in improving neck-related disability.

**Table 2.** Primary and secondary outcomes for the OG^a^ and the TG^b^.

Outcome and time point (wk)	OG (n=58), mean (SD)	TG (n=58), mean (SD)	Mean difference (TG − OG), (95% CI)	*P* value
Primary outcome				
NDI^c^ (%)				
4	26.9 (10.1)	24.1 (9.8)	−2.8 (−6.5 to 0.9)	.13
8	22.5 (9.7)	20.2 (9.3)	−2.3 (−5.8 to 1.2)	.20
12	21.9 (9.6)	19.1 (9.3)	−2.8 (−6.3 to 0.7)	.11
24	20.8 (9.2)	18.3 (10.8)	−2.5 (−6.2 to 1.2)	.15
Secondary outcomes				
NPRS^d^				
4	4.7 (1.4)	4.6 (1.3)	−0.1 (−0.6 to 0.4)	.69
8	4.2 (1.0)	4.0 (1.2)	−0.2 (−0.6 to 0.2)	.56
12	3.9 (1.3)	3.8 (1.0)	−0.1 (−0.5 to 0.3)	.64
24	3.6 (1.2)	3.3 (1.1)	−0.3 (−0.7 to 0.1)	.16
EQ-5D				
4	0.82 (0.18)	0.8 (0.17)	−0.02 (−0.08 to 0.04)	.53
8	0.82 (0.16)	0.84 (0.16)	+0.02 (−0.04 to 0.08)	.50
12	0.83 (0.17)	0.85 (0.15)	+0.02 (−0.04 to 0.08)	.50
24	0.85 (0.15)	0.86 (0.16)	+0.01 (−0.05 to 0.07)	.73
ROM^e^ (°)				
Flexion				
4	59.0 (7.9)	57.2 (7.6)	−1.8 (−4.6 to 1.1)	.21
8	60.7 (7.8)	62.3 (7.7)	+1.6 (−1.3 to 4.5)	.27
12	65.1 (8.2)	64.0 (8.1)	−1.1 (−4.1 to 1.9)	.47
24	63.2 (7.9)	65.5 (7.8)	+2.3 (−0.6 to 5.2)	.12
Extension				
4	59.1 (8.7)	61.0 (8.5)	+1.9 (−1.3 to 5.1)	.24
8	61.3 (8.6)	64.2 (8.4)	+2.9 (−0.2 to 6.0)	.07
12	63.1 (9.2)	66.2 (9.0)	+3.1 (−0.2 to 6.5)	.07
24	66.0 (9.0)	67.5 (8.8)	+1.5 (−1.8 to 4.8)	.37
Left lateral flexion				
4	38.1 (2.9)	39.0 (2.8)	+0.9 (−0.2 to 1.9)	.09
8	41.5 (2.9)	41.0 (3.1)	−0.5 (−1.6 to 0.6)	.37
12	42.0 (3.9)	42.1 (4.5)	+0.1 (−1.4 to 1.6)	.88
24	43.8 (3.7)	43.0 (2.5)	−0.8 (−2.0 to 0.4)	.18
Right lateral flexion				
4	38.2 (3.0)	39.2 (4.1)	+1.0 (−0.3 to 2.3)	.14
8	39.7 (3.8)	41.2 (3.6)	+1.5 (0.1 to 2.9)	.03
12	43.1 (4.1)	42.2 (3.4)	−0.9 (−2.3 to 0.5)	.20
24	43.0 (2.6)	43.2 (2.4)	+0.2 (−0.7 to 1.1)	.67
Left rotation				
4	58.3 (6.4)	60.0 (6.2)	+1.7 (−0.6 to 4.0)	.15
8	59.8 (6.3)	62.0 (6.0)	+2.2 (−0.1 to 4.5)	.06
12	62.0 (6.0)	64.0 (7.9)	+2.0 (−0.6 to 4.6)	.13
24	63.4 (6.8)	65.0 (6.6)	+1.6 (−0.9 to 4.1)	.20
Right rotation				
4	59.5 (7.1)	61.1 (7.0)	+1.6 (−1.0 to 4.2)	.22
8	61.0 (6.4)	62.0 (6.1)	+1.0 (−1.3 to 3.3)	.30
12	64.6 (6.3)	66.1 (6.1)	+1.5 (−0.8 to 3.8)	.19
24	65.2 (6.3)	67.0 (6.0)	+1.8 (−0.5 to 4.1)	.12

a OG: outpatient rehabilitation group.

b TG: telerehabilitation group.

c NDI: Neck Disability Index.

d NPRS: Numeric Pain Rating Scale.

e ROM: range of motion.

#### Secondary Outcomes

Baseline NPRS scores were comparable between the groups. No statistically significant between-group differences were observed at weeks 4, 8, 12, or 24 (Table 2). At week 24, the mean NPRS score had decreased from 5.6 to 3.6 in the OG and from 5.9 to 3.3 in the TG.

Baseline EQ-5D scores were comparable between the groups. No statistically significant between-group differences were observed at weeks 4, 8, 12, or 24 (Table 2). At week 24, the mean EQ-5D score increased from 0.69 to 0.85 in the OG and from 0.71 to 0.86 in the TG.

Baseline cervical ROM values were comparable between the groups. Flexion-extension, lateral flexion, and rotation improved in both groups during follow-up. Except for greater right lateral flexion in the TG at week 8 (between-group difference +1.5°, 95% CI 0.1°-2.9°; *P*=.03), no statistically significant between-group differences were observed across the remaining time points or movement planes (Table 2).

At week 24, the reductions in NPRS and increases in EQ-5D in both groups met the corresponding reported MCID thresholds, and improvements in most cervical ROM planes reached or exceeded the lower clinically meaningful threshold of 5°. Overall, both groups showed clinically meaningful and broadly comparable improvements in pain intensity, health-related quality of life, and cervical ROM.

### Harms

No intervention-related adverse events were documented in either group during the 24-week intervention.

### Ancillary Analyses

Exploratory post hoc responder analyses included all 116 randomized participants, with missing follow-up data treated as nonresponse (Table 3). At week 24, clinically meaningful improvement in the NDI was achieved by 53 of 58 (91.4%) participants in the TG and 51 of 58 (87.9%) participants in the OG (*P*=.76). For the NPRS, 53 of 58 (91.4%) participants in the TG and 47 of 58 (81.0%) participants in the OG met the responder criterion (*P*=.18). For the EQ-5D, 41 of 58 (70.7%) participants in the TG and 43 of 58 (74.1%) participants in the OG met the responder criterion (*P*=.84). Similar responder patterns were observed at weeks 4 and 12, with no statistically significant between-group differences in the NDI, NPRS, or EQ-5D at any reported time point.

For cervical ROM, responder rates were generally comparable between the groups. The TG had higher flexion responder rates than the OG at week 12 (19/58, 32.8% vs 7/58, 12.1%; *P*=.01) and week 24 (22/58, 37.9% vs 11/58, 19.0%; *P*=.04). In contrast, the OG had a higher responder rate for left lateral flexion at week 24 (38/58, 65.5% vs 26/58, 44.8%; *P*=.04). No statistically significant between-group differences were observed for the remaining cervical ROM comparisons. Because these analyses were exploratory and were not adjusted for multiple comparisons, the isolated differences in cervical ROM responder rates should be interpreted cautiously.

**Table 3.** Exploratory post hoc responder analyses for clinically meaningful improvement in NDI^a^, NPRS^b^, and EQ-5D, as well as threshold-based improvement in cervical ROM^c^.

Outcome^d^ and time point (wk)	TG^e^ responders (n=58), n (%)	OG^f^ responders (n=58), n (%)	*P* value
NDI			
4	50 (86.2)	45 (77.6)	.34
12	53 (91.4)	50 (86.2)	.56
24	53 (91.4)	51 (87.9)	.76
NPRS			
4	35 (60.3)	32 (55.2)	.71
12	49 (84.5)	45 (77.6)	.48
24	53/58 (91.4)	47 (81.0)	.18
EQ-5D			
4	34 (58.6)	36 (62.1)	.85
12	37 (63.8)	39 (67.2)	.85
24	41 (70.7)	43 (74.1)	.84
ROM			
Flexion			
4	15 (25.9)	11 (19.0)	.51
12	19 (32.8)	7 (12.1)	.01
24	22 (37.9)	11 (19.0)	.04
Extension			
4	28 (48.3)	22 (37.9)	.35
12	30 (51.7)	33 (56.9)	.71
24	39 (67.2)	37 (63.8)	.85
Left lateral flexion			
4	15 (25.9)	20 (34.5)	.42
12	27 (46.6)	29 (50.0)	.85
24	26 (44.8)	38 (65.5)	.04
Right lateral flexion			
4	21 (36.2)	14 (24.1)	.23
12	32 (55.2)	29 (50.0)	.71
24	34 (58.6)	25 (43.1)	.14
Left rotation			
4	32 (55.2)	21 (36.2)	.06
12	35 (60.3)	33 (56.9)	.85
24	46 (79.3)	37 (63.8)	.10
Right rotation			
4	38 (65.5)	30 (51.7)	.19
12	48 (82.8)	43 (74.1)	.37
24	48 (82.8)	46 (79.3)	.81

a NDI: Neck Disability Index.

b NPRS: Numeric Pain Rating Scale.

c ROM: range of motion.

d Responder thresholds were defined as a reduction of at least 10 percentage points for the NDI, a reduction of at least 2 points for the NPRS, an increase of at least 0.03 for the EQ-5D, and an increase of at least 5° for cervical ROM.

e TG: telerehabilitation group.

f OG: outpatient rehabilitation group.

## Discussion

### Interpretation

This study aimed to compare AI-enabled telerehabilitation with standard outpatient rehabilitation in patients with CNP. After 24 weeks, follow-up completion rates were 94.8% (55/58) in the TG and 91.4% (53/58) in the OG, with a slightly higher rate in the TG. For the primary outcome, no significant between-group difference in NDI improvement was observed, and the upper bound of the 95% CI at week 24 was below the prespecified noninferiority margin, demonstrating noninferiority of the TG. NDI scores decreased by 22.9 percentage points in the TG and 20.1 percentage points in the OG, both exceeding the 10-point threshold for clinically meaningful improvement. For the secondary outcomes, improvements in NPRS, EQ-5D, and cervical ROM were generally comparable between groups. At week 24, NPRS scores decreased by 2.6 and 2.0 points and EQ-5D scores increased by 0.15 and 0.16 in the TG and OG, respectively, meeting the MCIDs; most cervical ROM planes also showed clinically meaningful improvement. Because the cervical ROM analyses involved multiple exploratory comparisons without adjustment for multiplicity, the isolated significant findings should be interpreted cautiously. Overall, both interventions produced clinically meaningful and broadly comparable effects.

Previous studies have suggested that telerehabilitation may improve pain and disability in patients with cervical conditions and chronic neck pain [21-24,32-36]. However, previous trials generally used relatively short interventions of 6 to 8 weeks and compared telerehabilitation with unsupervised home exercise, ergonomic education, digital self-care, or in-person exercise [35,36]. Differences in intervention duration, comparator, and level of supervision have limited the available evidence on the longer-term effects of telerehabilitation [34-36]. Compared with these studies, this study used a multicenter, randomized noninferiority design; extended the intervention period to 24 weeks; and ensured that both groups received the same exercise content, dosage, and progression criteria, with therapist oversight provided in both groups. In addition to evaluating the effect of AI-enabled telerehabilitation on neck-related disability, this study assessed its effects on pain, health-related quality of life, and cervical ROM. These design features enabled a more direct and comprehensive evaluation of remotely delivered structured exercise over a longer period.

Both treatment programs were associated with less pain, better quality of life, and improved ability to carry out daily activities. As shown in Table 2, NDI scores fell markedly from baseline by week 4, while EQ-5D scores rose progressively in both groups, and these changes met the prespecified thresholds for clinical importance. Pain improvement occurred more gradually; however, by week 24, changes in NPRS reached the MCID in both groups, and gains in most cervical ROM planes were greater than 5°. Table 3 also shows high week-24 responder rates for NDI, NPRS, and EQ-5D in both groups, with generally comparable response patterns. Taken together, these results suggest that structured exercise may improve neck-related function and general health status relatively early, whereas pain and cervical mobility may continue to improve as training is sustained.

Exercise therapy is widely regarded as a central component of CNP management [12], yet its benefits depend on patients continuing the prescribed training regularly over time [37-41]. In the conventional outpatient setting, repeated clinic visits, transportation difficulties, work-related time constraints, and unequal availability of rehabilitation resources can make continued participation more difficult [18-20]. In addition, supervision is usually intermittent, so movement errors, problems with exercise progression, or other difficulties that arise outside the clinic may not be recognized or managed promptly, which may compromise continuity of care and long-term treatment effects [21,38].

Therefore, this study used the HRS, which combines a smartphone-based platform with movement sensors. The system was designed to extend exercise guidance, movement monitoring, and follow-up support into the home environment. Movement demonstrations, standardized instructions, sensor-based recognition, and real-time feedback helped participants complete strengthening and stretching exercises. Previous studies of internet-, smartphone-, and sensor-based telerehabilitation for cervical conditions and chronic neck pain have also reported improvements in pain and physical function [21,23,32-35].

The telerehabilitation system also enabled participants to receive timely guidance from physical therapists. Therapists remotely reviewed training records and movement performance, evaluated system-generated recommendations for progression or adjustment, and made the final exercise-prescription decisions. During the 24-week intervention, participants in the TG trained at home 3 times per week for approximately 30 minutes per session. Participants in the OG received the same exercise content, dosage, and progression framework under face-to-face outpatient supervision. Thus, the main difference between groups was the setting and mode of supervision rather than the exercise treatment itself. The findings showed that transferring training from the outpatient clinic to the home did not reduce clinical effectiveness when standardized exercise and therapist supervision were maintained. Sensor-based feedback may have supported exercise quality during home training, while remote therapist review maintained clinical oversight of exercise progression and adjustment [38,39]. These features may help explain why home-based telerehabilitation achieved outcomes comparable to outpatient rehabilitation [21,38].

Compared with the OG, the TG allowed participants to train at home around their daily schedules, reducing time spent on repeated appointments and hospital travel while providing greater flexibility [18-21,36]. The slightly higher week-24 completion rate in the TG suggests that home-based delivery may support continued participation and greater engagement in rehabilitation [39]. Less travel, waiting, and time away from work may also reduce time-related costs and other rehabilitation expenses [38,42]. Standardized exercise prescriptions, sensor-based feedback, and therapist review allowed participants to continue receiving professional guidance at home. Remote delivery may also extend therapist reach and ease pressure on outpatient rehabilitation resources [13-17,38]. Future studies should compare medical costs, resource use, and cost-effectiveness between the 2 models.

Overall, AI-enabled telerehabilitation allowed patients with CNP to continue structured exercise at home while receiving real-time feedback and professional supervision. In addition to maintaining clinical outcomes comparable to standard outpatient rehabilitation, this model may reduce the effects of geographic distance, transportation barriers, work commitments, and limited rehabilitation resources on continued treatment [18-21,38-40]. It may therefore serve as a practical alternative for patients who have difficulty attending regular face-to-face rehabilitation and provide a feasible approach to continuous, long-term CNP management [38,39].

### Limitations

This multicenter, randomized noninferiority trial was conducted at 2 hospitals in China and included 116 patients with CNP to compare AI-enabled telerehabilitation with standard outpatient rehabilitation over 24 weeks. First, because the interventions differed visibly in their delivery, participants and treating physical therapists were aware of group allocation, which may have introduced performance bias. To reduce this risk, outcome assessors and statistical analysts remained blinded. Adherence was measured using platform-generated training logs in the TG and attendance and prescription-completion records in the OG, limiting direct quantitative comparison. Future studies should use common adherence definitions and measures applicable to both delivery models. In addition, the exploratory cervical ROM analyses were not adjusted for multiple comparisons; therefore, isolated significant findings should be interpreted cautiously. Second, all participants were able to use a smartphone-based rehabilitation system. The delivery of telerehabilitation may still depend on age, digital literacy, acceptance of technology, and access to appropriate devices and reliable internet connections [39,43]. These barriers may be particularly relevant to older adults and digitally underserved populations [43]. Future research should therefore include patients with varying levels of digital ability and offer standardized device training, practical instructions, and continued technical support. Finally, no formal health economic evaluation was conducted, so the 2 rehabilitation models were not directly compared in terms of patient travel and time costs, health care resource use, therapist workload, or overall cost-effectiveness. Future studies should collect real-world resource-use data and perform cost-effectiveness or cost-utility analyses to better assess the economic value and practical scalability of AI-enabled telerehabilitation.

### Conclusions

In this multicenter, randomized noninferiority trial, AI-enabled telerehabilitation was noninferior to exercise-matched outpatient rehabilitation for improving neck-related disability over 24 weeks, with broadly comparable improvements in pain, health-related quality of life, and cervical ROM. The study’s innovation lies in applying sensor-based movement recognition, threshold rules, and therapist oversight to deliver the same structured exercise program at home for patients with CNP. Compared with previous studies that were generally shorter or used different comparators [21,23,34,35], this trial provides longer-term evidence for structured digital rehabilitation. Larger pragmatic studies should assess implementation, cost-effectiveness, and generalizability across broader populations and clinical settings. Overall, AI-enabled telerehabilitation may be a practical alternative for patients with CNP who have difficulty attending regular outpatient sessions.

## Supplementary material

10.2196/87487 Multimedia Appendix 1 Detailed 24-week exercise protocol for chronic nonspecific neck pain.

10.2196/87487 Checklist 1 CONSORT 2025 checklist.

10.2196/87487 Checklist 2 CONSORT-EHEALTH checklist.

## Abbreviations

CNP: chronic nonspecific neck pain
CONSORT: Consolidated Standards of Reporting Trials
CONSORT-EHEALTH: Consolidated Standards of Reporting Trials of Electronic and Mobile Health Applications and Online Telehealth Interventions
HRS: Healbone Rehabilitation System
MCID: minimal clinically important difference
NDI: Neck Disability Index
NPRS: Numeric Pain Rating Scale
OG: outpatient rehabilitation group
ROM: range of motion
TG: telerehabilitation group
