# Supplementary material for: AI-Enabled Telerehabilitation for Chronic Nonspecific Neck Pain: Multicenter Randomized Controlled Trial
Source: JMIR Form Res. 2026 Oct 15;10:e87487. doi: 10.2196/87487 (PMC13671386; doi:10.2196/87487)
Supplement: Supplementary file 1 [file formative-v10-e87487-s002.pdf]

CONSORT 2025 expanded checklist of detailed information to include when reporting a randomised trial

Section / Topic	No	CONSORT 2025 checklist item description	Detailed item description	Location in manuscript
**Title and abstract**				
Title and structured abstract	1a	Identification as a randomised trial	Use the word "randomised" in the title	Page 1.
	1b	Structured summary of the trial design, methods, results, and conclusions	● Specific objectives<br>● Trial design (e.g., parallel group, cluster) and framework (e.g., superiority, equivalence, non- inferiority, exploratory)<br>● Methods:<br>○ Eligibility criteria for participants and settings where the trial was conducted<br>○ Intervention(s) and comparator(s) intended for each group<br>○ Primary outcome(s)<br>○ How participants were allocated to interventions (e.g., centralised computer-generated randomisation)<br>○ Who was blinded after assignment to interventions (e.g., participants, care providers, outcome assessors)<br>● Results:<br>○ Number of participants randomised to each group<br>○ For the primary outcome, the number of participants analysed in each group<br>○ For the primary outcome, a result for each group and the estimated effect size and its precision<br>○ Important harms or unintended events for each group<br>● Conclusions:<br>○ General interpretation of the results<br>● Name of trial register and identification number<br>● Sources of funding<br>*Do not report information that does not appear in the body of the paper*	Pages 1–3.
**Open science**				
Trial registration	2	Name of trial registry, identifying number (with URL) and date of	● Name of registry<br>● Trial registry identifying number<br>● URL to registry record	Pages 3 and 6.

		registration		
			• Date of registration<br>• Whether the trial results are already publicly posted to the trial registry, as a preprint (with URL citation) or published articles (with citations)	
Protocol and statistical analysis plan	3	Where the trial protocol and statistical analysis plan can be accessed	• Where the protocol can be accessed with URL to its location (e.g., publication with DOI, repository such as Open Science Framework (OSF), trial registry, supplement to the trial report)<br>• Where the full statistical analysis plan can be accessed with URL to its location (e.g., publication with DOI, repository such as Open Science Framework, trial registry, supplement to the trial report)	Page 23.
Data sharing	4	Where and how the individual de-identified participant data (including data dictionary), statistical code and any other materials can be accessed	• What data and materials are shared, for example:<br>○ De-identified participant data, data dictionary, analytical code used to process the data<br>○ Materials associated with the intervention (e.g., handbook or video for non-pharmacological interventions)<br>• Where the data and materials are accessible (e.g., upon request, through a data sharing platform)<br>• Where the data and materials are accessible (e.g., upon request, through a data sharing platform)<br>• How the data and materials are shared (e.g., application process to access the data)<br>• If no sharing is planned, this should be clearly stated with an explanation	Pages 9–11 and 23; Multimedia Appendix 1.
Funding and conflicts of interest	5a	Sources of funding and other support (e.g., supply of drugs), and role of funders in the design, conduct, analysis and reporting of the trial	• Name of funder(s)<br>• Type of funding:<br>○ Direct monetary support<br>○ Indirect support (free trial drugs, equipment, or services such as statistical analysis or use of medical writers)<br>• Role of the funder(s) in the trial design, conduct, data analysis and reporting	Page 23.

	5b	Financial and other conflicts of interest of the manuscript authors	● Conflicts of interests of the trial manuscript authors, including:<br>○ Financial: salary support or grants; ownership of stock or options; honoraria (e.g., for advice, authorship, or public speaking); paid consultancy or service on advisory boards; and holders of patents or patents pending<br>○ Non-financial: academic commitments; personal or professional relationships; other affiliations with special interests or advocacy positions<br>● Any procedures to reduce the influence of conflicts of interest on the trial's design, conduct, analysis, or reporting<br>● If no conflicts of interest, this should be clearly stated	Page 24.
**Introduction**				
Background and rationale	6	Scientific background and rationale	● Importance of the research question<br>● Why a new trial is needed in the context of available evidence<br>○ Explanation of how the intervention might work<br>○ Justification of the choice of comparator<br>○ Evidence of the benefits and harms of the intervention<br>○ Reference to systematic review(s) of relevant trials where available	Pages 3–5.
Objectives	7	Specific objectives related to benefits and harms	● Trial objective(s) related to benefits and harms including:<br>○ Participants<br>○ Intervention(s)<br>○ Comparator(s)<br>○ Primary outcome(s)<br>○ Timepoint of primary outcome<br>● If the trial was designed using the estimands framework, the objectives should be defined in terms of this framework	Page 5.
**Methods**				
Patient and public involvement	8	Details of patient or public involvement in the design, conduct and reporting of the trial	● How patients and the public were involved at different trial stages (e.g., design, conduct, reporting)<br>● Who was involved (e.g., patients, carers, or members of the public)<br>● If no patient or public involvement, this should be stated	Page 6.

Trial design	9	Description of trial design including type of trial (e.g., parallel group, crossover), allocation ratio, and framework (e.g., superiority, equivalence, non-inferiority, exploratory)	● Type of trial design (e.g., parallel group)<br>● Conceptual framework (e.g., superiority, non-inferiority, or equivalence)<br>● Unit of randomisation (e.g., individual participant)<br>● Allocation ratio (e.g., 1:1)	Page 6.
Changes to trial protocol	10	Important changes to the trial after it commenced including any outcomes or analyses that were not prespecified, with reason	● Any changes to the original protocol after the trial commenced with timing and reasons, e.g., in randomisation ratio, eligibility criteria, interventions, outcomes (method of assessment, timepoint, changes of outcomes), target sample size, number of trial groups, duration of follow-up, analysis methods, trial conduct (such as dropping a site with poor data quality), any other important changes<br>● Any outcomes that were not prespecified<br>● Any analyses that were not prespecified	Pages 5-6, 11-12, and 17–19.
Trial setting	11	Settings (e.g., community, hospital) and locations (e.g., countries, sites) where the trial was conducted	● Location(s) where the trial was carried out (e.g., country, city)<br>● Setting of participant recruitment (e.g., primary or tertiary care; outpatient community or hospital clinic, inpatient unit)<br>● Number of sites	Pages 6–7.
	12a	Eligibility criteria for participants	● All inclusion and exclusion criteria<br>● Methods of recruitment (e.g., referral or self-selection; advertisements)	Pages 7 and Textbox 1.

Eligibility criteria	12b	If applicable, eligibility criteria for sites and for individuals delivering the interventions (e.g., surgeons, physiotherapists)	If applicable:<br>● Eligibility criteria for sites, for example the sites volume for the procedure<br>● Eligibility criteria for individuals delivering the interventions (e.g., surgeons, physiotherapists), such as professional qualifications, years in practice, skills, validation of specific training before trial initiation	Pages 6 and 8–10.
Intervention and comparator	13	Intervention and comparator with sufficient details to allow replication. If relevant, where additional materials describing the intervention and comparator (e.g., intervention manual) can be accessed	● Details of each intervention and comparator to allow replication including for example:<br>○ Components of the intervention and comparator<br>○ How they were administered<br>○ When and for how long they were administered<br>○ Any procedure for tailoring the intervention/comparator to individual participants<br>○ Any physical or informational materials used as part of the intervention/comparator (e.g., instruction manual) and where the materials can be accessed<br>○ Where appropriate, relevant concomitant care and interventions that are allowed (e.g., rescue interventions) or prohibited during the trial<br>○ Where appropriate, criteria used to guide modifications to the trial intervention/comparator (e.g., drug dose change in response to harms, participant request, or improving/worsening disease) and discontinuations of the trial intervention/comparator<br>● When the comparator group is "usual care":<br>○ Description of usual care<br>○ Whether the intervention group(s) also received usual care<br>● When and how fidelity of care providers and adherence of participants to the intervention/comparator protocols were assessed, if applicable<br>● Any strategies for improving fidelity of care providers and adherence of participants to the intervention/comparator protocols<br>● Where appropriate, prespecified definition for classifying participants as being treated as planned or not	Pages 7 and 8–10; Multimedia Appendix 1.

Outcomes	14	Pre-specified primary and secondary outcomes, including the specific measurement variable (e.g., systolic blood pressure), analysis metric (e.g., change from baseline, final value, time to event), method of aggregation (e.g., median, proportion), and time point for each outcome	• Which outcomes are primary and secondary as prespecified in the protocol<br>• Rationale for the choice of trial outcomes and whether they are part of a core outcome set<br>• For each outcome:<br>○ Specific variable measured (e.g., Beck Depression Inventory score) with definition where relevant (e.g., major bleeding was defined as fatal bleeding or symptomatic bleeding in a critical area or organ; all-cause mortality as per hospital database)<br>○ Analysis metric for each participant (e.g., change from baseline, end value, time-to-event)<br>○ Method of aggregation for each trial group (e.g., median, proportion with score > 2)<br>○ Timepoint of interest for analysis (e.g., 3 months)<br>○ Who assessed outcomes (patient, doctor, nurse, caregiver, other)	Page 5 and pages 10–12.
Harms	15	How harms were defined and assessed (e.g., systematically, non-systematically)	• For each systematically assessed harm (active/targeted surveillance):<br>○ Definition and measurement (e.g., name of a validated questionnaire)<br>○ Where appropriate, the metrics, method of aggregation and time point of interest for analysis (see item 14)<br>○ Procedures for harms assessment, including:<br>▪ Who did the assessment, and whether they were blinded to the allocated trial group<br>▪ Assessment time points and the overall time period for recording harms For each non-systematically assessed harm (passive surveillance):<br>○ How data were collected<br>○ Assessment time points and overall time period for recording harms<br>○ Process for coding each harm and grading its severity, including:<br>▪ Who did the coding and severity grading, and whether they were blinded to the allocated trial group<br>▪ Which coding and severity grading systems were used, if any<br>• For grouping of harms by seriousness, severity, body system, withdrawals (due to harms), and causality:<br>○ Definitions of grouping categories	Pages 10 and 17.

			○ Who did the grouping, and whether they were blinded to the allocated trial group	
Sample size	16a	How sample size was determined, including all assumptions supporting the sample size calculation	● Primary outcome on which the calculations are based<br>● Outcome values (e.g., proportion) assumed for each group, with rationale or supporting references<br>● Target difference in outcome values between trial groups (including common standard deviation for continuous outcomes), with rationale<br>● Statistical significance level or α (type I) error<br>● Statistical power or β (type II) error<br>● Any adjustments to account for e.g., missing data or non-adherence<br>● Target sample size per trial group<br>● Any software used for sample size calculation	Page 8.
	16b	Explanation of any interim analyses and stopping guidelines	Interim analyses:<br>● Whether interim analyses were conducted<br>● Whether interim analyses were pre-planned<br>● When they were conducted (timing and indications), and by whom<br>● Statistical methods<br>● Who had access to interim results, and whether they were blinded<br>● Whether an independent Data Monitoring Committee was involved<br>Stopping guidelines:<br>● Any criteria (statistical or non-statistical) used to inform decisions about early stopping or other adaptations (e.g., sample size re-estimation)<br>● Who made the decision to continue, stop, or modify the trial	Page 8.
Randomisation:				
Sequence generation	17a	Who generated the random allocation sequence and the method used	● Who generated the allocation sequence<br>● Method of sequence generation (e.g., computerised random number generator)<br>● Any software used for random sequence generation	Page 8.
	17b	Type of randomisation and details of any restriction (e.g., stratification, blocking and block size)	● Type of randomisation: simple versus restricted (e.g., blocked); fixed versus adaptive (e.g., minimisation, urn); and where relevant, the reasons for such choices<br>● Methods used for restriction:<br>○ Block randomisation:<br>▪ How blocks were generated (e.g., permuted block design with a computer random number generator)<br>. Block size(s)	Page 8.

			. Whether block size(s) was fixed or randomly varied<br>. Whether trialists were or became aware of block size(s)<br>○ Stratification:<br>. Factors of stratification (e.g., site, sex, disease stage)<br>○ Minimisation:<br>. Factors incorporated<br>. Whether a random element was used, and details	
Allocation concealment mechanism	18	Mechanism used to implement the random allocation sequence (e.g., central computer/telephone; sequentially numbered, opaque, sealed containers), describing any steps to conceal the sequence until interventions were assigned	● How the individuals enrolling participants were made unaware of the next trial group assignment in the random sequence (not to be confused with blinding)	Page 8.
Implementation	19	Whether the personnel who enrolled and those who assigned participants to the interventions had access to the random allocation sequence	● Who had access to the random allocation sequence<br>● Who enrolled participants<br>● Who assigned participants to interventions<br>● Whether personnel enrolling and assigning participants had access to the random allocation sequence.<br>● When individuals involved in sequence generation and allocation concealment are the same individuals involved in the implementation of assignment:<br>○ How and where the allocation list was stored<br>○ Any mechanisms to prevent those enrolling and assigning participants from accessing the list (e.g., allocation sequence was locked in a secure location)	Pages 7–8.

Blinding	20a	Who was blinded after assignment to interventions (e.g., participants, care providers, outcome assessors, data analysts)	Who was blinded to trial group assignments:<br>● Trial participants<br>● Care providers (i.e., those administering the intervention)<br>● Data collectors (those collecting data on the trial outcomes)<br>● Outcome assessors (i.e., those who determine if a participant experienced the outcome of interest), e.g., the participant for patient reported outcomes, the care provider, an independent researcher<br>● Data analysts performing the statistical analysis	Page 8.
	20b	If blinded, how blinding was achieved and description of the similarity of interventions	● Mechanism used to establish blinding (e.g., identical placebo, double-dummy)<br>● Any similarities or differences of the characteristics (e.g., appearance, taste) of the interventions being compared<br>● Any procedures intended to maintain blinding and reduce risk of unblinding, when appropriate<br>● Any procedures intended to evaluate blinding procedures (e.g., pre-trial testing of blinding procedures)<br>● Any known compromises in blinding (e.g., unblinding of any participants or caregivers during the trial)<br>● If done, any emergency unblinding with reasons and the procedure used	Pages 8–10 and 22.
Statistical methods	21a	Statistical methods used to compare groups for primary and secondary outcomes, including harms	● Statistical methods for each analysis:<br>○ Main analysis methods for statistical comparison<br>○ Any deviation from the statistical analysis plan<br>○ Distinction between prespecified and post-hoc analyses<br>○ Effect measure (e.g., absolute risk) with confidence intervals<br>○ Statistical significance level<br>● For Bayesian analysis: choices of priors, computational choices, details of any modelling, effect measure with credible intervals<br>● For adjusted analyses (if applicable):<br>○ Rationale for adjusted analyses<br>○ Whether adjusted analyses were pre-specified or post hoc<br>○ Choice of covariates adjusted for<br>○ Statistical methods (including how continuous covariates were handled)<br>● Methods to account for multiplicity, if applicable<br>● Software used for analyses	Pages 11–12, 17–18, and 22.

	21b	Definition of who is included in each analysis (e.g., all randomised participants), and in which group	● Who was included in the primary and other analyses (e.g., all randomised participants with either observed or imputed outcome data):<br>○ Any exclusions due to missing data or other reasons<br>● Trial group in which participants were analysed (e.g., as-randomised)	Pages 11–12 and 17–18.
	21c	How missing data were handled in the analysis	For each analysis:<br>● Assumption about the missing data mechanism (e.g., missing at random) with justification<br>● How missing data were handled (e.g., multiple imputation, model-based approaches), with justification<br>● Whether sensitivity analyses were conducted	Pages 11–12.
	21d	Methods for any additional analyses (e.g., subgroup and sensitivity analyses), distinguishing prespecified from post-hoc	● Whether additional analyses were pre-specified or post hoc (i.e., suggested by the data)<br>● Whether all additional analyses conducted are reported<br>For sensitivity analyses:<br>● Rationale<br>● Statistical methods<br>For subgroup analyses:<br>● Baseline variables explored<br>● Rationale<br>● Statistical methods (e.g., test of interaction)	Pages 11–12 and 17–18.
**Results**				

Participant flow, including flow diagram	22a	For each group, the numbers of participants who were randomly assigned, received intended intervention, and were analysed for the primary outcome	In a flow diagram, the number of participants:<br>● Evaluated for potential enrolment, if recorded<br>● Excluded before randomisation with reasons:<br>● Not meeting the inclusion criteria<br>● Declined to participate<br>● Other reasons<br>● Randomly assigned to each group<br>● Who received intervention as allocated, by trial group<br>● Who completed intervention as allocated, by trial group<br>● Who completed follow-up as planned, by trial group<br>● Included in the main analysis for the primary outcome, by trial group<br>Where appropriate (e.g., nonpharmacologic interventions):<br>● the number of care providers or centres performing the intervention in each group<br>● the number of participants treated by each care provider or in each centre	Pages 12–13 and Figure 2.
	22b	For each group, losses and exclusions after randomisation, together with reasons	In a flow diagram, the number of participants:<br>● Lost to follow up, by trial group<br>● Excluded from main analysis for the primary outcome, by trial group with reasons<br>The wording “protocol deviation” is not sufficiently explicit and exact reasons should be reported	Pages 12–13 and Figure 2.
Recruitment	23a	Dates defining the periods of recruitment and follow-up for outcomes of benefits and harms	● Start and completion date of participant recruitment<br>● Date when follow up ended<br>● Duration of follow-up (e.g., median, interquartile range, minimum, maximum)	Page 12.
	23b	If relevant, why the trial ended or was stopped	If relevant,<br>● Reason for stopping the trial before completion as planned (e.g., result of an interim analysis, lack of funding, difficulty in recruiting patients)<br>● Who made the decision to stop<br>● Role of funder in decision to stop	Not applicable; the trial was not stopped early.

Intervention and comparator delivery	24a	Intervention and comparator as they were actually administered (e.g., where appropriate, who delivered the intervention/comparator, whether participants adhered, whether they were delivered as intended [fidelity])	● Who actually delivered the intervention/comparator (number and expertise)<br>● How the intervention/comparator was actually administered<br>● What intervention/comparator was actually administered<br>● Participants' adherence to the intervention/ comparator<br>● Whether the intervention/comparator was delivered as intended (i.e., care provider's fidelity), where appropriate	Pages 8–10.
	24b	Concomitant care received during the trial for each group	In each trial group:<br>● Number and percentage of participants receiving the different relevant concomitant interventions (i.e., interventions that could have affected the outcome)<br>● Where relevant, the cumulative or average for each concomitant intervention taken over the trial period	Pages 7 and 12.
Baseline data	25	A table showing baseline demographic and clinical characteristics for each group	Baseline characteristics for participants in each group in a table detailing:<br>● For continuous variables:<br>○ Mean with standard deviation or median with percentiles (e.g., 25th, 75th), where appropriate<br>● For binary and categorical variables:<br>○ Numbers and percentages	Page 14, Table 1.
Numbers analysed,	26	For each primary and secondary outcome, by group:	For all planned primary and secondary outcomes, in each group:<br>● The number of participants included in analysis<br>● The number of participants with available data at the outcome time point	Pages 12 and 15–16; Table 2.

outcomes and estimation		● the number of participants included in the analysis<br>● the number of participants with available data at the outcome time point<br>● result for each group, and the estimated effect size and its precision (such as 95% confidence interval)<br>● for binary outcomes, presentation of both absolute and relative effect size	● Reasons for missing data<br>● For continuous outcomes:<br>○ Summary of the outcome in each group (e.g., mean with standard deviation)<br>○ Effect size (e.g., difference in means) and its precision (e.g., 95% confidence interval)<br>● For binary or time-to-event outcomes:<br>○ Summary of the outcome in each group (e.g., number of participants with the outcome event and denominator)<br>○ Both absolute (e.g., risk difference, difference in median survival time, difference of restricted mean survival time) and relative effect size (e.g., risk ratio, odds ratio, hazard ratio, ratio of restricted mean survival time) and its precision (e.g., 95% confidence interval)	Pages 12 and 14 –17;Table 2.
Harms	27	All harms or unintended events in each group	For each group preferably in a table with the number of participants at risk:<br>● Number of deaths<br>● Number of participants withdrawn due to harms<br>For the systematically assessed harms, non-systematically assessed harms, and serious harms in each group preferably in a table with the number of participants at risk:<br>● Number of participants with at least one harm event<br>● Number of events, if appropriate<br>● If no adverse events were identified, this should be stated.<br>● Where appropriate<br>○ The estimated effect size and its precision (95% confidence interval)<br>○ For binary outcomes/time-to-event outcomes, both absolute and relative effects	Page 17.
Ancillary analyses	28	Any other analyses performed, including subgroup and sensitivity analyses, distinguishing pre-	● Results for all other analyses performed<br>● Describe which were pre-specified and which were post-hoc analyses	Pages 17–19 and Table 3.

		specified from post-hoc		
Discussion				
Interpretation	29	Interpretation consistent with results, balancing benefits and harms, and considering other relevant evidence	• Brief summary of the trial results, balancing both benefits and harms of the intervention(s)<br>• How the trial results relate to existing evidence (e.g. systematic review)<br>• Avoid overinterpretation ('spin')	Pages 19–22.
Limitations	30	Trial limitations, addressing sources of potential bias, imprecision, generalisability, and, if relevant, multiplicity of analyses	• Any methodological limitations and, if relevant, any methods used to minimise or mitigate them<br>• Any imprecision in the results<br>• Generalisability of the results	Pages 22–23.

Citation: Hopewell S, Chan AW, Collins GS, Hróbjartsson A, Moher D, Schulz KF, et al. CONSORT 2025 Statement: updated guideline for reporting randomised trials. BMJ. 2025; 388:e081123.https://dx.doi.org/10.1136/bmj-2024-081123
© 2025 Hopewell et al. This is an Open Access article distributed under the terms of the Creative Commons Attribution License (https://creativecommons.org/licenses/by/4.0/), which permits unrestricted use, distribution, and reproduction in any medium, provided the original work is properly cited.

*We strongly recommend reading this statement in conjunction with the CONSORT 2025 Explanation and Elaboration for important clarifications on all the items. We also recommend reading relevant CONSORT extensions. Seewww.consort-spirit.org.
