# Supplementary material for: AI-Enabled Telerehabilitation for Chronic Nonspecific Neck Pain: Multicenter Randomized Controlled Trial
Source: JMIR Form Res. 2026 Oct 15;10:e87487. doi: 10.2196/87487 (PMC13671386; doi:10.2196/87487)
Supplement: Supplementary file 3 [file formative-v10-e87487-s001.docx]

## ****Exercise Protocol****

Training frequency and duration: three sessions per week, approximately 30 minutes per session, for a total of 24 weeks.

## ****Phase 1 - 0 to 4 weeks - pain control and postural reset****

### ****Goals****

- reduce neck/shoulder pain and stiffness
- restore gentle cervical mobility within pain-free range
- activate deep neck flexors and scapular stabilizers; improve postural awareness

### ****Precautions****

- avoid sudden rotation/overextension; stay within pain-free range
- maintain neutral head/neck; normal breathing throughout
- stop if dizziness, numbness, or radiating pain occurs

### ****Rehabilitation Exercise Table****

| **Exercise** | **Description** |
| --- | --- |
| Supported chin tuck (supine) | Lie on back with small pillow/towel roll to support neck curve. Gently draw chin inward to form a “double chin”. Hold 3 seconds, relax. |
| Cervical AROM (gentle) | In neutral sitting, gently perform flexion, extension, rotation and side-bending within pain-free range, 5–8 reps each direction. |
| Isometric neck contraction | Press palm against forehead/side/back of head without moving the head. Hold 5 seconds, repeat. |
| Scapular retraction | Sit or stand, gently squeeze shoulder blades together for 5 seconds, then relax. |
| Pectoral/upper thoracic stretch | Stand facing corner, arms on walls, lean forward slightly to stretch chest/upper back. |
| “W” supported arm slide (supine) | In supine, elbows bent in “W”, slide arms outward and return while keeping shoulder blades engaged. |
| Marching in place (low intensity) | Stand and alternately lift knees to comfortable height with controlled rhythm. |

## ****Phase 2 – 4 to 12 weeks – strengthening and neuromuscular control****

### ****Goals****

- build neck–shoulder strength and endurance
- enhance coordination and dynamic postural control
- progress range of motion as tolerated

### ****Precautions****

- avoid lifting excessively heavy objects; progress gradually
- keep head over spine; avoid forward-head posture and shrugging
- perform 2–3 sets based on tolerance

### ****Rehabilitation Exercise Table****

| **Exercise** | **Description** |
| --- | --- |
| Wall slide (W→Y) | Back against wall, arms in “W”, slowly slide up to “Y”, then return. |
| Wall press | Face the wall, press gently with palms, engaging shoulder stabilizers. |
| Standing T raise | Lift arms sideways to shoulder height to form “T”, pause, then lower slowly. |
| Scapular squeeze (endurance) | Squeeze shoulder blades, hold 5 seconds, relax; repeat. |
| Towel isometric row | Hold towel ends and pull outward, maintaining isometric scapular retraction. |
| Wall angel | With back/arms against wall, slide arms up/down in a controlled motion. |
| Cervical retraction (seated/standing) | Retract chin gently to align head over spine; hold 2–3 seconds. |
| Wall ball side bend (light) | Soft ball between head and wall; apply gentle side pressure and hold. |
| Marching with arm rhythm | March in place while coordinating bilateral arm swings. |

## ****Phase 3 – 12 to 24 weeks – advanced strengthening and functional maintenance****

### ****Goals****

- increase muscular endurance and functional capacity of neck–shoulder complex
- reinforce dynamic postural stability for daily/work tasks
- establish sustainable home routine for long-term symptom control

### ****Precautions****

- remain symptom-guided; avoid provocative end-range loading
- progress resistance/time before adding range or complexity
- maintain neutral spine; avoid breath-holding

### ****Rehabilitation Exercise Table****

| **Exercise** | **Description** |
| --- | --- |
| Band-resisted cervical retraction | Anchor a light band at forehead level; retract chin against resistance, hold 3–5 seconds, control return. |
| Band cervical extension/flexion (light–moderate) | Anchor band behind (extension) or in front (flexion); perform small-range controlled reps while maintaining neutral posture. |
| Band lateral isometrics | Band anchored laterally at head level; maintain neutral while resisting side pull for 5–8 seconds each side. |
| Serratus wall slide with band | Elastic band around forearms; forearms on wall, slide upward while keeping ribs down and neck neutral. |
| Standing Y/T raises (with or without band) | Raise arms to “Y” and “T” patterns at shoulder height, emphasizing scapular control. |
| Band row (standing) | Anchor band at chest level; pull elbows back, squeeze shoulder blades, slow return. |
| Dynamic posture hold | Stand tall, gently retract chin and lower ribs, hold 10–15 seconds with diaphragmatic breathing. |
| March with overhead reach | Alternate knee lifts while reaching arms overhead; maintain head–neck alignment. |
| Functional carry (light, 1–3 kg each hand) | Walk 20–30 meters holding light objects at sides, shoulders depressed, head neutral. |
